# Admission White Blood Cell Count and Neutrophil‐to‐Lymphocyte Ratio for Early Risk Stratification after Spontaneous Intracerebral Hemorrhage: A Retrospective Cohort Study

**DOI:** 10.1002/brb3.71786

**Published:** 2026-10-13

**Authors:** Guangyan Li, Gang Wang, Yuhang Hui, Xiaoyang Xiong, Haoyu Wang, Boru Hou

**Affiliations:** ^1^ Department of Neurosurgery Lanzhou University Second Hospital Lanzhou China

**Keywords:** discharge outcome, ICH score, internal validation, neutrophil‐to‐lymphocyte ratio, spontaneous intracerebral hemorrhage, white blood cell count

## Abstract

**Objective:**

To evaluate the adjusted associations and incremental prognostic value of admission white blood cell count (WBC) and neutrophil‐to‐lymphocyte ratio (NLR) for unfavorable discharge outcome after spontaneous intracerebral hemorrhage (sICH).

**Methods:**

Consecutive adults with CT‐confirmed sICH admitted to a tertiary academic hospital in northwestern China from August 2021 through August 2025 were retrospectively reviewed. After eligibility screening, 764 patients formed the study cohort. Admission WBC and log_2_‐transformed NLR were analyzed as continuous exposures; the primary outcome was discharge modified Rankin Scale (mRS) score ≥ 3. Multivariable models were benchmarked against the ICH score and a clinical severity model. Internal validation used 1000 bootstrap resamples. A three‐level outcome‐derived WBC‐NLR burden classification was treated as exploratory and evaluated with 1000 nested bootstrap resamples.

**Results:**

The primary complete‐case analysis included 752 patients, of whom 358 (47.6%) had an unfavorable discharge outcome. Each 1 × 10^9^/L increase in WBC was associated with higher odds of unfavorable outcome (adjusted odds ratio [OR], 1.116; 95% CI, 1.035–1.204; *p* = 0.004), and each doubling of NLR was associated with an adjusted OR of 2.187 (95% CI, 1.687–2.835; *p* < 0.001). Adding WBC and NLR increased the ICH score AUC from 0.757 to 0.848 (ΔAUC, 0.091; *p* < 0.001) and the clinical model AUC from 0.875 to 0.909 (ΔAUC, 0.034; *p* < 0.001). The optimism‐corrected AUC of the full model was 0.900, with a corrected calibration slope of 0.932 and Brier score of 0.129. The nested‐bootstrap‐corrected AUC was 0.776 for the exploratory burden‐only model and 0.899 after adding clinical covariates.

**Conclusions:**

Admission WBC and NLR remained associated with unfavorable discharge outcome after adjustment and improved discrimination beyond established severity measures. They may support preliminary short‐term risk assessment, whereas the outcome‐derived burden categories require external validation. The discharge endpoint should not be interpreted as a substitute for 90‐day functional outcome.

## Graphical abstract

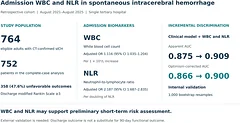

## 1 Introduction

Spontaneous intracerebral hemorrhage (sICH) is among the most disabling forms of stroke and remains a neurological emergency with substantial early mortality and long‐term dependency. Contemporary American and European guidelines emphasize rapid severity assessment and coordinated neurocritical care (Greenberg et al. 2022; Steiner et al. 2025), and a high‐level clinical review summarizes the continuing prognostic importance of hematoma‐related primary and secondary injury (Sheth 2022). Early prognosis is strongly related to consciousness level, hematoma volume, intraventricular extension, infratentorial origin, and age, which form the basis of the widely used ICH score (Hemphill et al. 2001; Hemphill et al. 2009). Nevertheless, patients with similar anatomic injury may experience different short‐term courses, suggesting that systemic responses to the hemorrhage may provide additional prognostic information.

Inflammation contributes to secondary brain injury after ICH through leukocyte recruitment, blood‐brain barrier disruption, cytokine release, oxidative stress, and perihematomal edema (Chen et al. 2021; Zille et al. 2022). WBC provides a broad measure of circulating leukocytosis, whereas NLR integrates neutrophil‐predominant inflammatory activation with relative lymphopenia and acute stress‐related immune dysregulation. Systematic reviews, meta‐analyses, and clinically important cohorts have linked higher admission WBC or NLR to mortality and functional disability after ICH (Guo and Zou 2024; Shi et al. 2022; Guo et al. 2025; Yu et al. 2016; He et al. 2023; Agnihotri et al. 2011; Lattanzi et al. 2016; Tao et al. 2017; Qin et al. 2019; Lattanzi et al. 2018). However, reported thresholds vary markedly, and associations may overlap with initial hemorrhage severity.

Outcome‐derived biomarker thresholds can discard information, increase residual confounding, and produce optimistic estimates when cutoff selection and evaluation are performed in the same cohort (Altman and Royston 2006; Royston et al. 2006). The clinical contribution of inflammatory markers is therefore best judged through transparent prediction‐model reporting, comparison with established severity measures, and internal validation rather than apparent sample performance alone (Collins et al. 2015; Steyerberg et al. 2001).

To address these issues, continuous WBC and log_2_‐transformed NLR were analyzed as the primary exposures; their incremental value was benchmarked against the ICH score and a conventional clinical severity model, and bootstrap internal validation was performed. The outcome‐derived three‐level burden classification was retained only as an explicitly exploratory analysis and evaluated with nested bootstrap validation. Dose‐response patterns, overlap with conventional severity indicators, in‐hospital complications, and an exploratory surgery interaction were also examined.

## 2 Materials and Methods

### 2.1 Study Design and Population

This single‐center retrospective cohort study followed the STROBE reporting recommendations (von Elm et al. 2007). The study was conducted at Lanzhou University Second Hospital, a tertiary academic referral center serving Gansu Province and neighboring areas in northwestern China. Consecutive adults admitted to the Departments of Neurosurgery or Neurology from August 2021 through August 2025 were screened. Inclusion criteria were age ≥ 18 years, CT‐confirmed spontaneous intraparenchymal hemorrhage, initial head CT performed within 48 h of symptom onset, admission laboratory testing and key imaging assessment, and an available discharge mRS score. Exclusion criteria were traumatic hemorrhage; isolated subdural, subarachnoid, or epidural hemorrhage; hemorrhage secondary to aneurysm, vascular malformation, tumor, or hemorrhagic transformation of ischemic stroke; documented active infection before admission; severe immune or hematologic disease; long‐term immunosuppressive therapy; and missing primary outcome data.

Of 780 screened records, seven patients younger than 18 years and nine records with noneligible hemorrhage type or location were excluded, leaving 764 eligible patients. Exploratory inflammatory burden could be classified in 761 patients with available WBC and NLR values. The primary complete‐case model included 752 patients after excluding 6 with missing hematoma location, 3 with missing NLR, and 3 with missing initial hematoma volume.

### 2.2 Data Collection and Variable Definitions

Demographic characteristics, medical history, admission vital signs, laboratory values, imaging findings, treatment, in‐hospital complications, and discharge outcome were extracted from the electronic medical record using standardized variable definitions. The first complete blood count obtained after hospital admission and before operative treatment was used. WBC was expressed as ×10^9^/L. NLR was calculated as the absolute neutrophil count divided by the absolute lymphocyte count. The exact onset‐to‐blood‐sampling interval was not consistently recorded and therefore could not be analyzed as a continuous or stratified covariate.

Admission neurological severity was measured using the GCS. Initial hematoma volume was obtained from the first CT examination using the institutional radiology measurement recorded in the medical record. Hematoma location was classified as basal ganglia, thalamic, lobar, ventricular, cerebellar, or brainstem. Intraventricular hemorrhage was coded as present when ipsilateral or bilateral ventricular extension was documented. Midline shift was analyzed in millimeters. Treatment was grouped as conservative management or surgery; minimally invasive procedures and open craniotomy were combined for the exploratory interaction analysis.

The ICH score was calculated using its original components: GCS 3–4 (2 points), GCS 5–12 (1 point), age ≥ 80 years (1 point), hematoma volume ≥ 30 mL (1 point), intraventricular hemorrhage (1 point), and infratentorial origin (1 point) (Hemphill et al. 2001).

### 2.3 Outcome and Exploratory Inflammatory Burden

The primary outcome was unfavorable functional status at hospital discharge, defined as mRS ≥ 3. A sensitivity analysis used mRS ≥ 4. Discharge mRS was abstracted from the functional assessment documented by the treating team. Standardized 90‐day mRS was not systematically collected in this retrospective cohort and was not reconstructed because incomplete post‐discharge records would create substantial misclassification. Functional status can continue to change between early follow‐up and 90 days after ICH, so the discharge endpoint was interpreted as a short‐term hospital outcome rather than a surrogate for standardized 90‐day recovery (Coleman et al. 2024).

For the exploratory burden analysis, final‐sample cutoffs were selected by maximizing the Youden index: 9.510 × 10^9^/L for WBC and 7.954 for NLR. Low burden required both markers below their cutoffs; moderate burden required elevation of either marker; and high burden required elevation of both markers. Because the thresholds were outcome‐derived, all category‐based estimates were explicitly considered exploratory.

### 2.4 In‐Hospital Complications

Pulmonary infection, tracheostomy, intracranial infection, hypoproteinemia, electrolyte disturbance, deep‐vein thrombosis, and pleural effusion were assessed during the index hospitalization. Pulmonary infection required a physician‐recorded diagnosis supported by compatible respiratory findings and chest imaging, consistent with the emphasis on standardized clinical and radiographic criteria in stroke‐associated pneumonia research (Smith et al. 2015). Intracranial infection required a physician‐recorded diagnosis supported by cerebrospinal fluid, microbiological, or imaging findings when obtained. Deep‐vein thrombosis and pleural effusion required imaging confirmation, and tracheostomy was identified from procedure records. Hypoproteinemia required serum total protein below the institutional lower reference limit or a corresponding physician diagnosis. Electrolyte disturbance required an abnormal sodium, potassium, calcium, magnesium, or phosphate value accompanied by clinical documentation. Only complications with reproducible definitions were included; no composite cardiovascular outcome was analyzed. Post‐stroke infections are common and are associated with adverse outcomes, but the present complication analyses were descriptive rather than causal (Westendorp et al. 2011).

### 2.5 Missing Data and Sample‐Size Considerations

Missingness was limited to hematoma location in six patients (0.8%), NLR in three (0.4%), and initial hematoma volume in three (0.4%); these records were mutually exclusive. Because missingness for every primary model variable was below 1%, the primary multivariable analyses used complete cases. No formal a priori sample‐size calculation was performed because all eligible consecutive records within the study period were included. The full model contained 14 non‐intercept parameters and 358 outcome events, corresponding to approximately 25.6 events per parameter.

### 2.6 Statistical Analysis

Continuous variables are reported as medians and interquartile ranges and were compared using the Kruskal–Wallis test. Categorical variables are reported as counts and percentages and were compared using Pearson chi‐square or Fisher exact tests when appropriate. All tests were two‐sided, with a prespecified significance level of .05. *p* values were formatted according to journal requirements.

Admission WBC and log_2_‐transformed NLR were the primary continuous exposures, thereby avoiding the information loss and residual confounding associated with primary categorization of continuous predictors (Altman and Royston 2006; Royston et al. 2006). Covariates were selected because they are established early prognostic factors in sICH and were available at or shortly after admission. Multivariable logistic regression adjusted for age, sex, hypertension, GCS score, initial hematoma volume, intraventricular hemorrhage, midline shift, and hematoma location. NLR odds ratios therefore represent the association per doubling. Multicollinearity was assessed using generalized variance inflation factors. Dose‐response relationships were evaluated using restricted cubic spline logistic models with four knots; overall and nonlinear associations were assessed with Wald chi‐square tests.

Incremental predictive value was evaluated by comparing the ICH score with ICH score plus WBC and NLR and a clinical severity model with the same model plus WBC and NLR. Discrimination was quantified using AUCs and 95% CIs. Nested models were compared using paired DeLong and likelihood‐ratio tests. Calibration was assessed using the calibration intercept, calibration slope, Brier score, grouped observed‐versus‐predicted probabilities, and bootstrap‐corrected calibration curves. Decision‐curve analysis evaluated net benefit across threshold probabilities (Vickers and Elkin 2006).

Internal validation used 1000 bootstrap resamples, an efficient approach for estimating optimism in logistic prediction models (Steyerberg et al. 2001). Optimism was estimated as the mean difference between performance in each bootstrap sample and performance after applying the bootstrap‐fitted model to the original sample. For the outcome‐derived burden classification, 1000 nested bootstrap resamples repeated cutoff selection, category assignment, and model fitting within each resample.

Sensitivity analyses used mRS ≥ 4, winsorization of WBC and NLR at the first and 99th percentiles, and restriction to conservatively treated patients. The burden‐by‐surgery interaction was tested using a multiplicative term; stratified surgery estimates were considered exploratory and noncausal.

All numerical analyses were performed in R version 4.5.1 using rms 8.1–1, pROC 1.19.0.1, ggplot2 4.0.3, dplyr 1.2.1, broom 1.0.13, car 3.1–5, and gtsummary 2.5.1. Figure rendering and document assembly were independently checked against the exported model results. Statistical reporting followed SAMPL recommendations (Lang and Altman 2015).

### 2.7 Use of Generative Artificial Intelligence

A generative artificial‐intelligence tool assisted with code debugging and document preparation. It did not determine eligibility, variable definitions, model selection, statistical decisions, or interpretation. All scripts were executed by the authors, and all numerical results were checked against exported model outputs. No unavailable outcome, including 90‐day mRS, was generated or imputed.

### 2.8 Ethics

The study was approved by the Ethics Committee of Lanzhou University Second Hospital (Approval No. 2025A‐1073). The requirement for informed consent was waived because of the retrospective design and use of de‐identified clinical data.

## 3 Results

### 3.1 Cohort and Baseline Characteristics

The eligible cohort included 764 patients; 761 had both WBC and NLR available for exploratory burden classification, and 752 constituted the primary complete‐case cohort. Among the 752 complete cases, 358 (47.6%) had an unfavorable discharge outcome. Among the 761 classified patients, 262 were in the low, 189 in the moderate, and 310 in the high burden group. Unfavorable outcome occurred in 14.1%, 49.2%, and 75.5%, respectively. The participant flow is shown in Figure 1.

**FIGURE 1:**
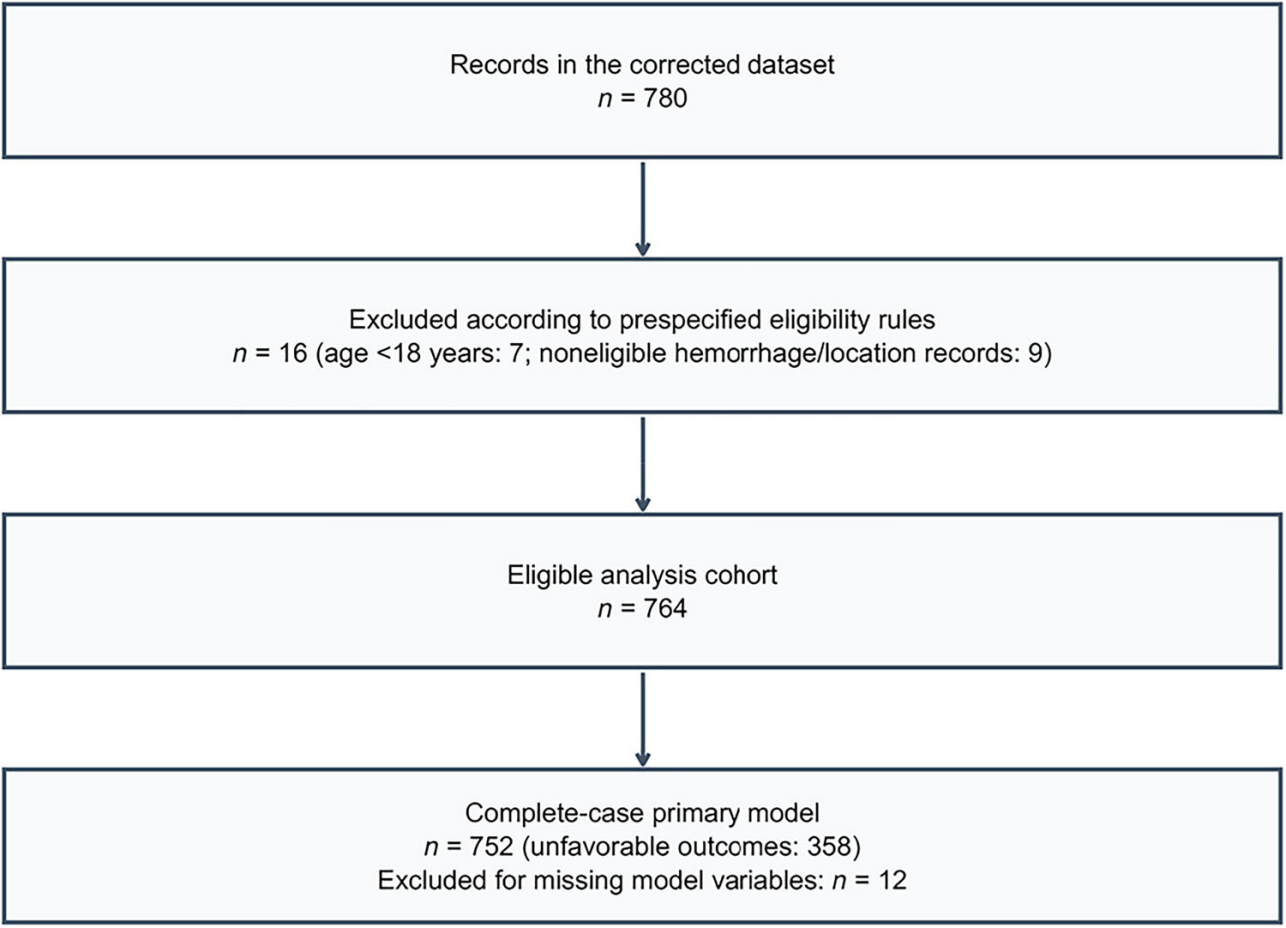
Participant flow and analytical populations. Among 780 screened records, seven patients younger than 18 years and nine records with noneligible hemorrhage type or location were excluded. The eligible cohort comprised 764 patients. Twelve patients were excluded from the complete‐case primary model because of missing hematoma location, NLR, or initial hematoma volume, resulting in 752 patients, including 358 with an unfavorable discharge outcome.

Age and sex distributions were similar across burden groups. Increasing burden was accompanied by lower GCS scores, larger hematoma volumes, more intraventricular hemorrhage and midline shift, and greater use of surgery. Hypertension increased from 69.8% in the low group to 81.9% in the high group (Table 1; Figure 2).

**TABLE 1:** Baseline characteristics according to exploratory WBC‐NLR inflammatory burden.

**Characteristic**	**Overall *N* = 761**	**Low *N* = 262**	**Moderate** ***N* = 189**	**High *N* = 310**	***p* value**
Age, years	57.00 (51.00, 66.00)	58.50 (52.00, 67.00)	58.00 (52.00, 67.00)	57.00 (51.00, 66.00)	*p* = 0.21
Male sex	391 (51.4%)	137 (52.3%)	93 (49.2%)	161 (51.9%)	*p* = 0.79
Hypertension	577 (75.8%)	183 (69.8%)	140 (74.1%)	254 (81.9%)	*p* = 0.003
GCS score	11.00 (9.00, 14.00)	13.00 (11.00, 15.00)	12.00 (9.00, 14.00)	9.00 (7.00, 12.00)	*p* < 0.001
WBC, ×10^9^/L	9.47 (7.22, 12.20)	6.81 (5.56, 7.77)	8.78 (7.83, 9.88)	12.78 (10.93, 14.99)	*p *< 0.001
NLR	8.88 (4.50, 13.97)	3.63 (2.69, 5.21)	9.45 (6.68, 12.96)	13.79 (10.54, 19.00)	*p* < 0.001
Intraventricular hemorrhage	189 (24.8%)	41 (15.6%)	49 (25.9%)	99 (31.9%)	*p* < 0.001
Basal ganglia location	420 (55.6%)	140 (54.1%)	92 (48.7%)	188 (61.2%)	*p* = 0.02*
Thalamic location	66 (8.7%)	19 (7.3%)	23 (12.2%)	24 (7.8%)	
Lobar location	166 (22.0%)	63 (24.3%)	41 (21.7%)	62 (20.2%)	
Ventricular location	41 (5.4%)	11 (4.2%)	14 (7.4%)	16 (5.2%)	
Cerebellar location	35 (4.6%)	10 (3.9%)	13 (6.9%)	12 (3.9%)	
Brainstem location	27 (3.6%)	16 (6.2%)	6 (3.2%)	5 (1.6%)	
Midline shift, mm	0.00 (0.00, 1.01)	0.00 (0.00, 0.00)	0.00 (0.00, 0.00)	0.00 (0.00, 6.67)	*p* < 0.001
Initial hematoma volume, mL	14.60 (5.24, 31.44)	7.93 (3.32, 17.01)	13.35 (5.07, 28.72)	27.36 (10.91, 42.75)	*p* < 0.001
Surgical treatment	321 (42.2%)	55 (21.0%)	76 (40.2%)	190 (61.3%)	*p* < 0.001
Unfavorable discharge outcome	364 (47.8%)	37 (14.1%)	93 (49.2%)	234 (75.5%)	*p* < 0.001

*Note*: Values are median (interquartile range) or *n* (%). *p* values compare all three groups. *p value for the overall hematoma‐location distribution. Burden groups were defined using outcome‐derived thresholds and should be interpreted as exploratory.

Recommended placement: Results, Section 3.1 (Cohort and baseline characteristics). Among 761 patients with both WBC and NLR available, age and sex distributions were similar across the three exploratory burden groups. In contrast, increasing inflammatory burden was accompanied by a higher prevalence of hypertension, lower GCS scores, larger initial hematoma volumes, more intraventricular hemorrhage and midline shift, and a higher proportion receiving surgical treatment. The proportion with unfavorable discharge outcome increased from 14.1% in the low‐burden group to 49.2% in the moderate‐burden group and 75.5% in the high‐burden group.

**FIGURE 2:**
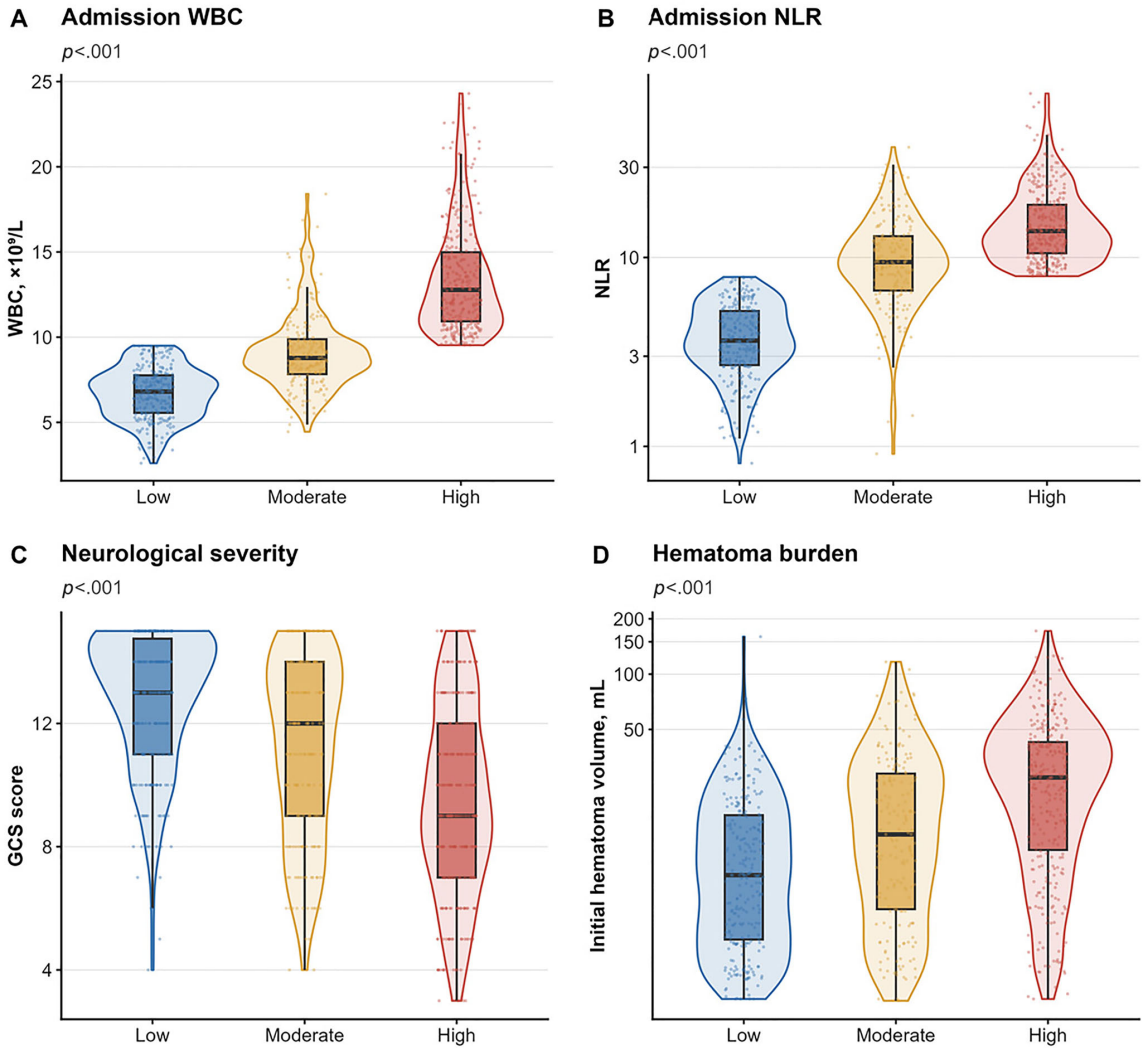
Distributions of admission inflammatory markers, neurological severity, and hematoma burden across exploratory WBC‐NLR inflammatory burden groups. Violin plots show the distributions, boxes indicate medians and interquartile ranges, and points represent individual patients. NLR is displayed on a logarithmic scale, and initial hematoma volume on a pseudo‐logarithmic scale. *p* values were calculated using the Kruskal–Wallis test. Because the WBC and NLR thresholds were selected in relation to the study outcome, the burden groups should be interpreted as exploratory.

### 3.2 Primary Continuous‐Marker Associations

In the fully adjusted complete‐case model, each 1 × 10^9^/L increase in WBC was associated with an 11.6% increase in the odds of an unfavorable discharge outcome (OR, 1.116; 95% CI, 1.035–1.204; *p* = 0.004). Each doubling of NLR was associated with more than a twofold increase in the odds of an unfavorable outcome (OR, 2.187; 95% CI, 1.687–2.835; *p* < 0.001) (Table 2; Figure S2). Hypertension and lower GCS scores were also associated with unfavorable outcome. The association of hematoma volume was attenuated after simultaneous adjustment for correlated severity variables.

**TABLE 2:** Adjusted associations of continuous inflammatory markers and exploratory burden categories with unfavorable discharge outcome.

**Analysis**	**Exposure**	**Adjusted OR (95% CI)**	***p* value**	***n***
Primary continuous analysis	WBC, per 1 × 10^9^/L	1.116 (1.035–1.204)	*p* = 0.004	752
Primary continuous analysis	NLR, per doubling	2.187 (1.687–2.835)	*p* < 0.001	752
Exploratory burden analysis	Moderate vs. low burden	5.344 (3.068–9.307)	*p* < 0.001	752
Exploratory burden analysis	High vs. low burden	12.241 (7.004–21.395)	*p* < 0.001	752

*Note*: All models adjusted for age, sex, hypertension, GCS score, initial hematoma volume, intraventricular hemorrhage, midline shift, and hematoma location. NLR was log_2_‐transformed; therefore, its OR represents the change in odds associated with a doubling of NLR.

Recommended placement: Results, Section 3.2 (Primary continuous‐marker associations), with the burden‐category rows referenced again in Section 3.5. In the fully adjusted complete‐case analysis of 752 patients, each 1 × 10^9^/L increase in admission WBC was associated with an 11.6% increase in the odds of an unfavorable discharge outcome (OR, 1.116; 95% CI, 1.035–1.204; *p* = 0.004). Each doubling of NLR was associated with more than a twofold increase in the odds of an unfavorable outcome (OR, 2.187; 95% CI, 1.687–2.835; *p* < 0.001). In the exploratory burden analysis, the adjusted ORs were 5.344 for moderate versus low burden and 12.241 for high versus low burden.

### 3.3 Model Performance and Internal Validation

The inflammatory‐marker‐only model had an AUC of 0.818. The ICH score had an apparent AUC of 0.757, which increased to 0.848 after adding WBC and NLR (ΔAUC, 0.091; DeLong *p* < 0.001; likelihood‐ratio *p* < 0.001). The clinical severity model AUC increased from 0.875 to 0.909 after adding WBC and NLR (ΔAUC, 0.034; DeLong *p* < 0.001; likelihood‐ratio *p* < 0.001). After 1000 bootstrap resamples, the full model had an optimism‐corrected AUC of 0.900, corrected calibration intercept of ‐0.005, calibration slope of 0.932, and Brier score of 0.129 (Table 3; Figure 3). Decision‐curve analysis suggested a modest increase in net benefit across clinically relevant thresholds, but this finding remains internal and requires external confirmation.

**TABLE 3:** Model performance and bootstrap internal validation.

**Model**	**Apparent AUC (95% CI)**	**Corrected AUC**	**Apparent brier**	**Corrected slope**
Inflammatory markers	0.818 (0.788–0.849)	—	0.173	—
ICH score	0.757 (0.725–0.789)	0.757	0.195	1.003
ICH score + WBC/NLR	0.848 (0.820–0.875)	0.847	0.158	0.993
Clinical model	0.875 (0.851–0.900)	0.866	0.143	0.939
Clinical model + WBC/NLR	0.909 (0.889–0.929)	0.900	0.122	0.932

*Note*: Corrected AUC and calibration slope were estimated using 1000 bootstrap resamples. A higher AUC indicates better discrimination; a lower Brier score indicates better overall probabilistic accuracy; a calibration slope closer to 1 indicates less overfitting. “‐” indicates that a bootstrap‐corrected estimate was not presented for that model.

Recommended placement: Results, Section 3.3 (Model performance and internal validation). The inflammatory‐marker‐only model had an AUC of 0.818. The apparent AUC of the ICH score increased from 0.757 to 0.848 after WBC and NLR were added (ΔAUC, 0.091; DeLong *p* < 0.001; likelihood‐ratio *p* < 0.001). The clinical model AUC increased from 0.875 to 0.909 after WBC and NLR were added (ΔAUC, 0.034; DeLong *p* < 0.001; likelihood‐ratio *p* < 0.001). After 1000 bootstrap resamples, the optimism‐corrected AUC of the full clinical model was 0.900, with a corrected calibration slope of 0.932 and a corrected Brier score of 0.129.

**FIGURE 3:**
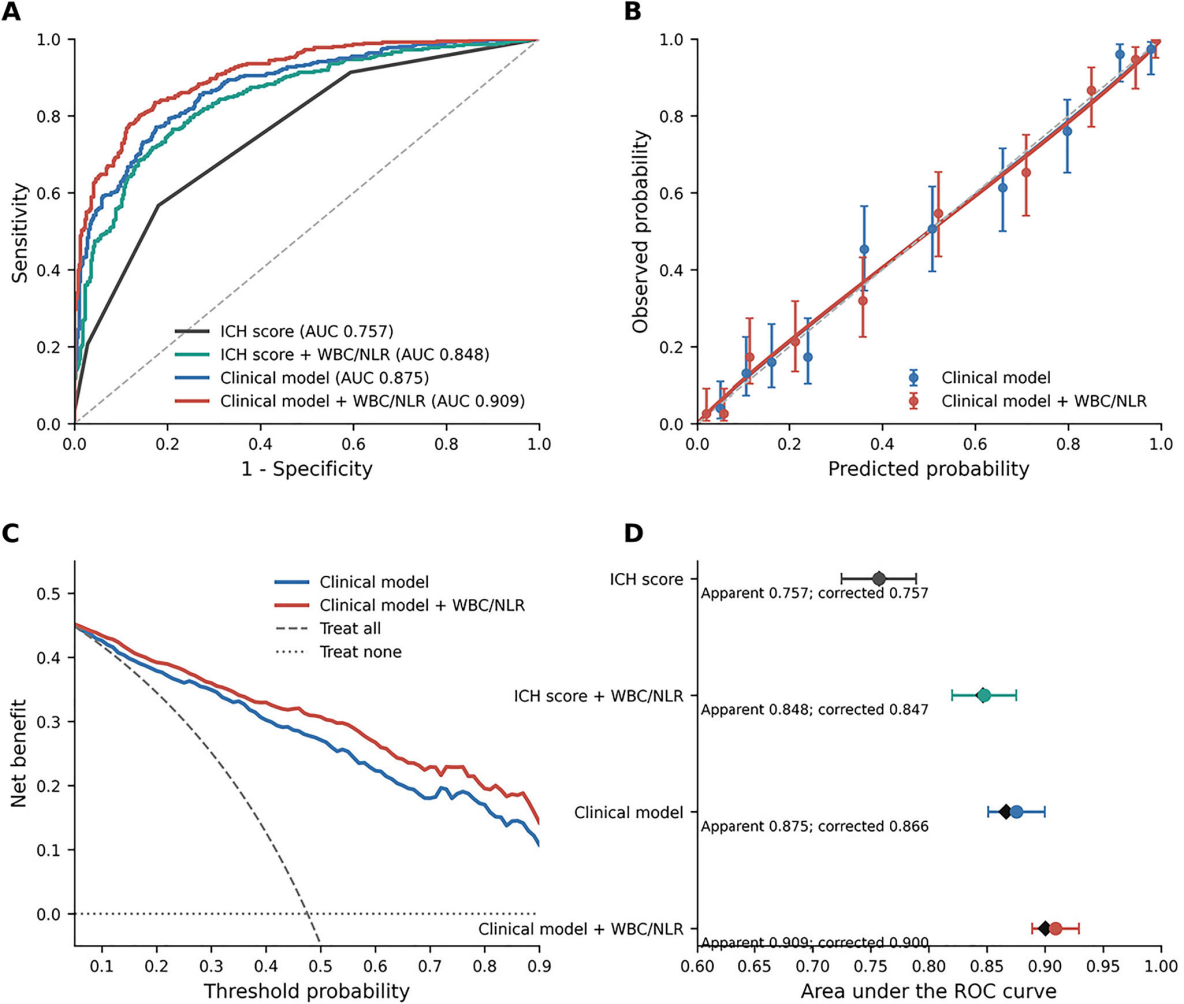
Model discrimination, calibration, clinical utility, and internal validation. (A) Receiver operating characteristic curves for the ICH score, ICH score plus WBC and NLR, clinical severity model, and clinical severity model plus WBC and NLR. (B) Grouped observed outcome proportions with Wilson 95% confidence intervals and bootstrap‐corrected calibration curves for the clinical models. (C) Decision‐curve analysis comparing the net benefit of the clinical model with and without WBC and NLR. (D) Apparent AUCs with 95% confidence intervals and optimism‐corrected AUCs derived from 1000 bootstrap resamples.

### 3.4 Dose‐Response and Overlap With Severity

Restricted‐spline analyses supported positive overall associations for WBC (overall *p* = 0.015) and NLR (overall *p* < 0.001). There was no strong evidence of nonlinearity for WBC (*p* = 0.89) or NLR (*p* = 0.19), indicating broadly monotonic relationships within the observed range (Figure 4). WBC and log_2_(NLR) were correlated (Spearman *ρ* = 0.63); log_2_(NLR) was inversely correlated with GCS (*ρ* = −0.49) and positively correlated with hematoma volume (*ρ* = 0.41). Nevertheless, adjusted generalized variance inflation factors were low (1.07–1.32), arguing against severe multicollinearity (Figure S3; Table S1).

**FIGURE 4:**
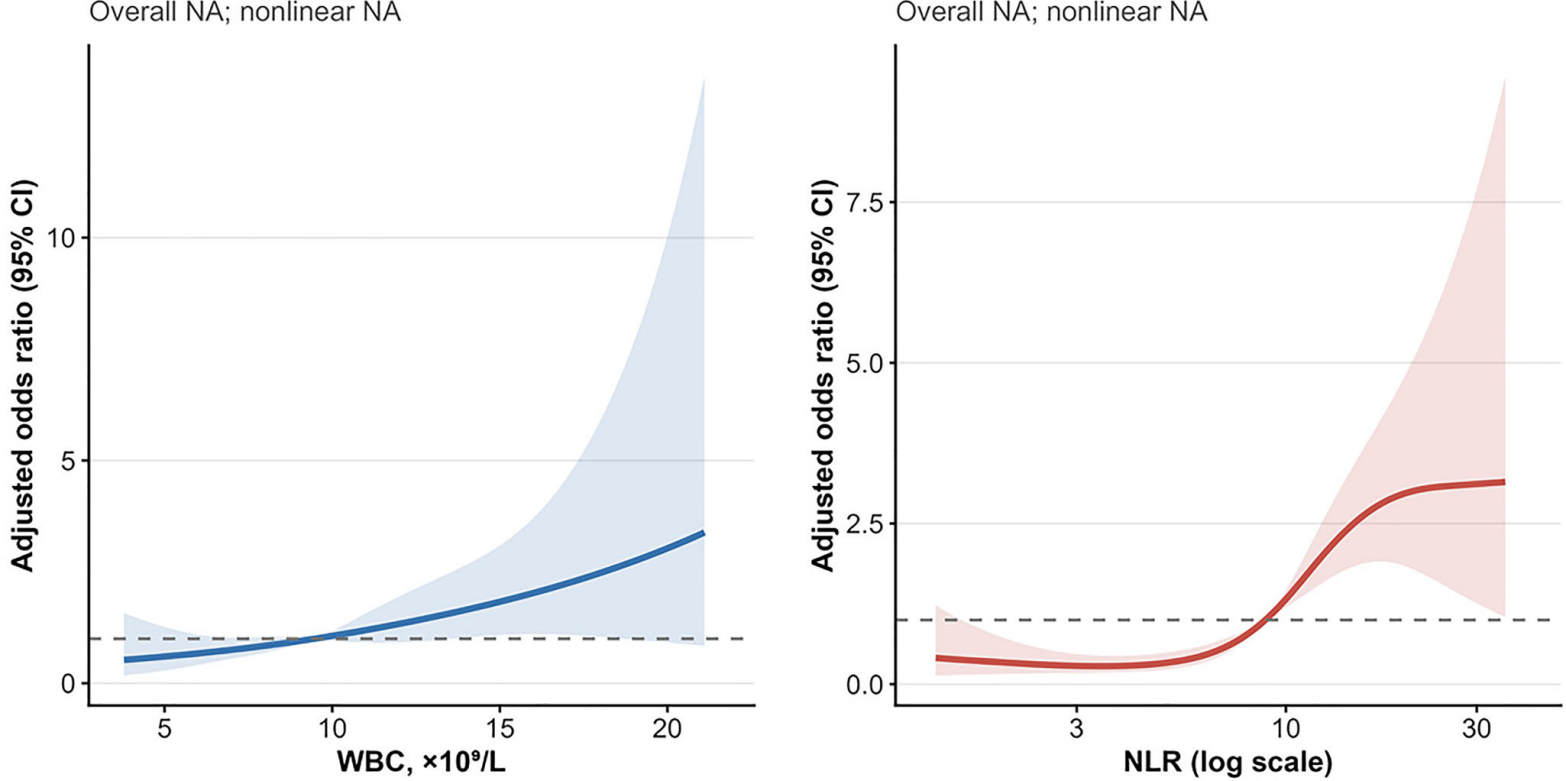
Adjusted restricted cubic spline associations of admission WBC and NLR with unfavorable discharge outcome. (A) WBC. (B) NLR. Solid lines indicate adjusted odds ratios, and shaded areas indicate 95% confidence intervals. The horizontal dashed line represents an odds ratio of 1. Models were adjusted for age, sex, hypertension, Glasgow Coma Scale score, initial hematoma volume, intraventricular hemorrhage, midline shift, hematoma location, and the alternate inflammatory marker. NLR is displayed on a logarithmic scale. Overall and nonlinear *p*‐values were obtained from Wald chi‐square tests.

### 3.5 Exploratory Outcome‐Derived Burden

Compared with the low burden group, the adjusted OR was 5.344 (95% CI, 3.068–9.307; *p* < 0.001) for moderate burden and 12.241 (95% CI, 7.004–21.395; *p* < 0.001) for high burden. Because these categories were defined using the study outcome, the magnitudes should not be interpreted as transportable risk estimates. In nested bootstrap validation, the burden‐only model AUC decreased from 0.784 to 0.776 after optimism correction, and the burden‐plus‐clinical‐covariate model decreased from 0.910 to 0.899. Bootstrap‐selected thresholds varied around the final‐sample values, further supporting cautious interpretation (Table S2; Figure S4).

### 3.6 In‐Hospital Complications

The proportions of pulmonary infection, tracheostomy, intracranial infection, hypoproteinemia, electrolyte disturbance, deep‐vein thrombosis, and pleural effusion increased across burden groups (Table 4; Figure 5). For example, pulmonary infection occurred in 41.6%, 52.9%, and 71.0% of the low, moderate, and high groups, respectively. These descriptive patterns may reflect both greater initial injury and subsequent systemic vulnerability; no causal direction was inferred.

**TABLE 4:** Major in‐hospital complications across exploratory burden groups.

**Complication**	**Low**	**Moderate**	**High**
Pulmonary infection	109/262 (41.6%)	100/189 (52.9%)	220/310 (71.0%)
Tracheostomy	9/262 (3.4%)	20/189 (10.6%)	65/310 (21.0%)
Intracranial infection	20/262 (7.6%)	20/189 (10.6%)	49/310 (15.8%)
Hypoproteinemia	36/262 (13.7%)	37/189 (19.6%)	82/310 (26.5%)
Electrolyte disturbance	53/262 (20.2%)	51/189 (27.0%)	104/310 (33.5%)
Deep‐vein thrombosis	10/262 (3.8%)	10/189 (5.3%)	31/310 (10.0%)
Pleural effusion	12/262 (4.6%)	14/189 (7.4%)	32/310 (10.3%)

*Note*: Data are *n*/*N* (%). Complications were assessed during the index hospitalization using the prespecified clinical and laboratory definitions. The table is descriptive and does not imply that inflammatory burden caused the complications.

Recommended placement: Results, Section 3.6 (In‐hospital complications). The proportions of pulmonary infection, tracheostomy, intracranial infection, hypoproteinemia, electrolyte disturbance, deep‐vein thrombosis, and pleural effusion increased across the low‐, moderate‐, and high‐burden groups. Pulmonary infection showed the largest absolute gradient, increasing from 41.6% to 52.9% and 71.0%, respectively. Tracheostomy increased from 3.4% in the low‐burden group to 21.0% in the high‐burden group.

**FIGURE 5:**
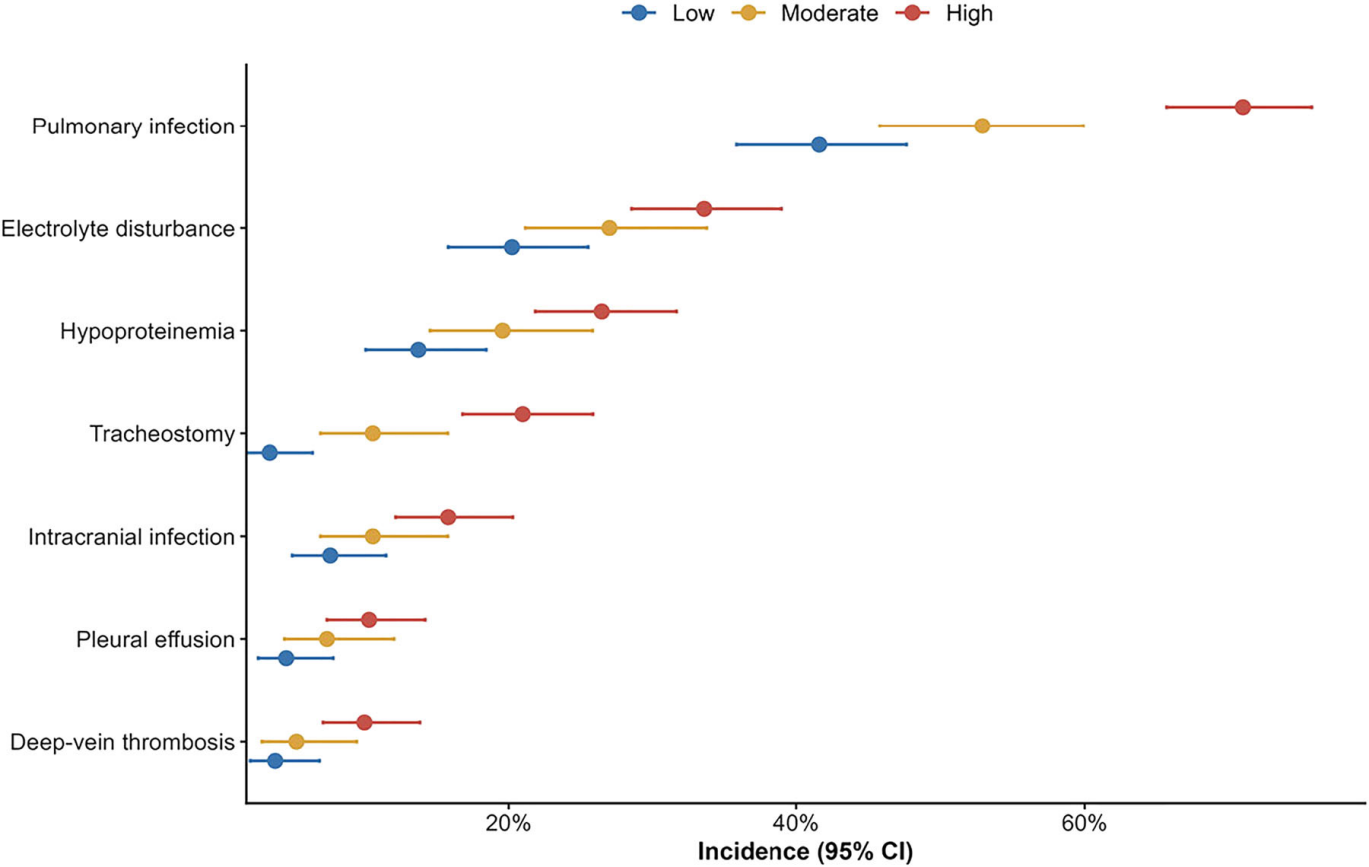
Proportions of patients with major in‐hospital complications across exploratory inflammatory burden groups. Points indicate observed proportions, and horizontal bars indicate Wilson 95% confidence intervals. Complications were ascertained during the index hospitalization according to the prespecified clinical and laboratory definitions.

### 3.7 Sensitivity and Surgery Analyses

Associations remained directionally consistent after winsorization and restriction to conservatively treated patients. For the alternative outcome of mRS ≥ 4, NLR remained associated with outcome (OR per doubling, 2.017; 95% CI, 1.537–2.646; *p* < 0.001), whereas the WBC estimate was attenuated (OR, 1.067; 95% CI, 0.995–1.145; *p* = 0.07) (Figure S6; Table S4). The burden‐by‐surgery interaction was not significant (*p* = 0.31). Although the moderate‐burden stratum showed an adjusted OR of 3.63 for surgery versus conservative treatment, the confidence interval was wide and the overall interaction was null. The surgery analysis was therefore moved to the supplement and should not be interpreted as evidence of differential treatment benefit (Figure S7; Table S3).

## 4 Discussion

Admission WBC and NLR provided prognostic information beyond conventional severity measures in this sICH cohort. The strongest evidence came from continuous‐marker models and benchmark comparisons: adding WBC and NLR increased the ICH score AUC by 0.091 and the clinical model AUC by 0.034. Bootstrap validation showed modest optimism in the full model, with an internally corrected AUC of 0.900 and calibration slope of 0.932. These findings support association and incremental discrimination within this cohort, but not immediate clinical implementation.

The results are consistent with systematic reviews, meta‐analyses, and major cohorts linking neutrophilia, lymphopenia, WBC, and NLR to mortality and poor functional outcome after ICH (Guo and Zou 2024; Shi et al. 2022; Guo et al. 2025; Yu et al. 2016; He et al. 2023; Agnihotri et al. 2011; Lattanzi et al. 2016; Tao et al. 2017; Qin et al. 2019; Lattanzi et al. 2018). The NLR estimate was stronger than the WBC estimate, which is biologically plausible because NLR combines acute neutrophil‐predominant activation with relative lymphocyte suppression. Neutrophils contribute to protease release, oxidative injury, blood‐brain barrier dysfunction, and perihematomal edema, whereas lymphopenia may reflect stress‐related immunosuppression (Chen et al. 2021; Zille et al. 2022).

Inflammation is closely intertwined with hemorrhage severity. The observed correlations between NLR, GCS, and hematoma volume indicate that stress leukocytosis and reverse causation are plausible. Low variance inflation factors show that the model was numerically estimable, but they do not establish biological independence or causality. The appropriate interpretation is that WBC and NLR retained statistical associations after adjustment, not that they define a distinct causal phenotype.

Outcome‐derived threshold selection and evaluation in the same sample can inflate discrimination and effect estimates and can sacrifice information contained in continuous measurements (Altman and Royston 2006; Royston et al. 2006; Collins et al. 2015; Steyerberg et al. 2001). Continuous markers were therefore used for the primary analyses, the three‐level categories were explicitly labeled “exploratory,” and threshold selection was repeated within every nested bootstrap sample. The modest decrease in AUC after correction indicates limited but nonzero optimism. External validation remains essential because published NLR thresholds vary across populations, sampling times, outcome definitions, and case mix (Guo and Zou 2024; Shi et al. 2022; Guo et al. 2025).

Benchmarking against the ICH score is clinically relevant. The ICH score was designed for simple early severity stratification and has been prospectively validated (Hemphill et al. 2001; Hemphill et al. 2009). The improvement after adding WBC and NLR suggests that routine inflammatory markers may complement rather than replace clinical and imaging predictors. Whether the small gain beyond a detailed clinical model produces meaningful changes in decisions requires prospective validation, standardized sampling, and formal impact analysis consistent with contemporary prediction‐model reporting principles (Collins et al. 2015).

Higher inflammatory burden was accompanied by more infections, tracheostomy, nutritional abnormalities, thrombosis, and pleural effusion. Stroke‐associated infection is common and is linked to adverse outcomes, while consensus recommendations emphasize standardized diagnostic criteria, particularly for pneumonia (Smith et al. 2015; Westendorp et al. 2011). These associations may identify patients requiring closer respiratory, infectious, nutritional, and thromboembolic surveillance, but they may also be consequences of greater neurological injury, invasive treatment, prolonged hospitalization, or immobility. Complication analyses were descriptive and should not be interpreted as mediation or causation.

The surgery findings remain exploratory. Randomized trials such as ENRICH and MISTIE III emphasize the importance of careful patient selection, technique, timing, location, and achieved hematoma reduction (Pradilla et al. 2024; Hanley et al. 2019). In retrospective practice, surgery is strongly confounded by indication. The nonsignificant interaction and wide stratum‐specific intervals do not support using inflammatory burden to select patients for surgery.

This study has limitations. First, the retrospective single‐center design limits transportability and permits residual confounding and information bias. Second, standardized 90‐day mRS was not available. Functional status can continue to improve between early assessment and 90 days after ICH (Coleman et al. 2024), and discharge mRS is influenced by length of stay, discharge destination, rehabilitation access, and withdrawal or limitation of care. Treatment‐limitation decisions can also affect the apparent performance of prognostic models after ICH (Zahuranec et al. 2010). The present findings therefore concern short‐term hospital status only. Third, only the first admission inflammatory measurement was analyzed, so dynamic trajectories were unavailable. Fourth, the timing of blood sampling relative to symptom onset was not sufficiently granular for time‐stratified analysis. Fifth, complete‐case analysis was reasonable because missingness was low, but it assumes that excluded observations did not introduce material bias. Sixth, the exploratory burden cutoffs were internally outcome‐derived and require external validation. Finally, observational associations cannot distinguish a contributory inflammatory pathway from stress responses caused by more severe hemorrhage.

## 5 Conclusion

Admission WBC and NLR remained associated with unfavorable discharge outcome after adjustment and improved discrimination when added to the ICH score and conventional clinical severity variables. Their low cost and availability make them promising adjuncts for preliminary short‐term risk assessment. However, the three‐level outcome‐derived burden classification remains exploratory, the observed associations do not establish causality, and external validation with standardized sampling and 90‐day or longer‐term functional outcomes is required before clinical use.

## Author Contributions

**Guangyan Li**: conceptualization, methodology, software, data curation, formal analysis, investigation, visualization, and original drafting. **Gang Wang**: validation, resources, and project administration. **Yuhang Hui and Xiaoyang Xiong**: investigation and resources. **Haoyu Wang**: data verification, imaging‐data review, and manuscript revision. **Boru Hou**: conceptualization, supervision, funding acquisition, project administration, and review and editing. All authors read and approved the final manuscript.

## Ethics Statement

This study was approved by the Ethics Committee of Lanzhou University Second Hospital (Approval No. 2025A‐1073). The requirement for informed consent was waived because of the retrospective design.

## Funding

This work was supported by the Scientific Research Project of Traditional Chinese Medicine of Gansu Province (Grant No. GZKZ‐2024‐28) and the Gansu Provincial Natural Science Foundation Project (Grant No. 25JRRA614). The funders had no role in study design; data collection, analysis, or interpretation; manuscript preparation; or the decision to submit the work.

## Conflicts of Interest

The authors declare no conflicts of interest.

## Supporting information




**Supplementary Material**: brb371786‐sup‐0001‐SuppMat.docx

## Data Availability

De‐identified data may be available from the corresponding author upon reasonable request, subject to institutional data security and ethics requirements.
