# Supplementary material for: Admission White Blood Cell Count and Neutrophil‐to‐Lymphocyte Ratio for Early Risk Stratification after Spontaneous Intracerebral Hemorrhage: A Retrospective Cohort Study
Source: Brain Behav. 2026 Oct 13;16(10):e71786. doi: 10.1002/brb3.71786 (PMC13659932; doi:10.1002/brb3.71786)
Supplement: Supplementary file 1 [file BRB3-16-e71786-s001.docx]

Supplementary Material

Admission White Blood Cell Count and Neutrophil-to-Lymphocyte Ratio for Early Risk Stratification After Spontaneous Intracerebral Hemorrhage: A Retrospective Cohort Study

# Supplementary Methods

Definitions of complications, missing-data handling, sensitivity analyses, nested bootstrap validation, and the exploratory surgery analysis were consistent with the main Methods. Pulmonary infection required a physician-recorded diagnosis with compatible clinical features and chest imaging; intracranial infection required a physician-recorded diagnosis supported by cerebrospinal-fluid, microbiological, or imaging findings when obtained; deep-vein thrombosis and pleural effusion required imaging confirmation; tracheostomy was based on procedure records; hypoproteinemia required total protein below the institutional lower reference limit or a corresponding diagnosis; and electrolyte disturbance required a laboratory abnormality with clinical documentation. In every nested bootstrap sample, WBC and NLR cutoffs were reselected before burden classification and model fitting. Surgery analyses were exploratory and were not interpreted causally.

## Supplementary Table S1. Generalized variance inflation factors.

| Variable | Adjusted GVIF |
| --- | --- |
| wbc | 1.228 |
| log2_nlr | 1.164 |
| age | 1.072 |
| gender | 1.037 |
| hypertension | 1.082 |
| gcs | 1.095 |
| volume | 1.317 |
| ivh_binary | 1.156 |
| midline_shift | 1.203 |
| position | 1.071 |

## Supplementary Table S2. Nested bootstrap validation of the outcome-derived burden classification.

| Model | Apparent AUC | Mean optimism | Corrected AUC | Successful resamples | WBC cutoff, ×10⁹/L | NLR cutoff |
| --- | --- | --- | --- | --- | --- | --- |
| Burden only | 0.784 | 0.009 | 0.776 | 1000 | 9.510 | 7.954 |
| Burden + clinical covariates | 0.910 | 0.011 | 0.899 | 1000 | 9.510 | 7.954 |

## Supplementary Table S3. Exploratory surgery associations by inflammatory burden stratum.

| Burden stratum | n | Events | Surgery, n | Adjusted OR | 95% CI lower | 95% CI upper | P value |
| --- | --- | --- | --- | --- | --- | --- | --- |
| Low | 258 | 36 | 53 | 1.21 | 0.38 | 3.84 | P=.74 |
| Moderate | 189 | 93 | 76 | 3.63 | 1.18 | 11.12 | P=.02 |
| High | 305 | 229 | 187 | 1.36 | 0.52 | 3.54 | P=.53 |

## Supplementary Table S4. Sensitivity analyses of continuous WBC and NLR.

| Analysis | Adjusted OR | 95% CI lower | 95% CI upper | P value | n | Marker |
| --- | --- | --- | --- | --- | --- | --- |
| Primary: mRS ≥3 | 1.116 | 1.035 | 1.204 | P=.004 | 752 | WBC |
| Primary: mRS ≥3 | 2.187 | 1.687 | 2.835 | P<.001 | 752 | NLR, per doubling |
| Alternative outcome: mRS ≥4 | 1.067 | 0.995 | 1.145 | P=.07 | 752 | WBC |
| Alternative outcome: mRS ≥4 | 2.017 | 1.537 | 2.646 | P<.001 | 752 | NLR, per doubling |
| Winsorized WBC/NLR | 1.116 | 1.035 | 1.204 | P=.004 | 752 | WBC |
| Winsorized WBC/NLR | 2.200 | 1.694 | 2.856 | P<.001 | 752 | NLR, per doubling |
| Conservative treatment only | 1.138 | 1.025 | 1.264 | P=.02 | 436 | WBC |
| Conservative treatment only | 1.900 | 1.362 | 2.651 | P<.001 | 436 | NLR, per doubling |

## Supplementary Table S5. Analytical sample size and events per parameter.

| Item | Value |
| --- | --- |
| Eligible cohort | 764 |
| Primary complete-case cohort | 752 |
| Unfavorable outcomes in primary cohort | 358 |
| Number of non-intercept model parameters | 14 |
| Events per parameter | 25.6 |

Supplementary Figure S1. Missingness among the 764 eligible patients. Only variables used in the primary model are shown.

Supplementary Figure S2. Fully adjusted associations of admission WBC and NLR with unfavorable discharge outcome. NLR was log₂-transformed, so the estimate represents the association per doubling.

Supplementary Figure S3. Spearman correlations among WBC, log₂(NLR), GCS score, initial hematoma volume, and midline shift.

Supplementary Figure S4. Distribution of WBC and NLR cutoffs selected across 1000 nested bootstrap samples. Cutoff selection was repeated within every resample.

Supplementary Figure S5. Distribution of discharge modified Rankin Scale scores across exploratory inflammatory burden groups.

Supplementary Figure S6. Sensitivity analyses using an alternative outcome of mRS ≥4, winsorized inflammatory markers, and restriction to conservatively treated patients.

Supplementary Figure S7. Exploratory association between surgery and unfavorable discharge outcome across inflammatory burden strata. The overall interaction was not statistically significant (P=.31); estimates should not be interpreted causally.
